# Toxicity of pyroligneous extract to *Folsomia candida* Willem, 1902 (Collembola: Isotomidae) in Tropical Artificial Soil

**DOI:** 10.1007/s11356-026-37996-2

**Published:** 2026-07-08

**Authors:** Ariane Sommer Rebolho, Vanessa Mignon Dalla Rosa, Nathalie Caroline Hirt Kessler, Flávia Alves Pereira, Dinéia Tessaro

**Affiliations:** 1 Federal Technological University of Paraná, Postal Code, Road to Boa Esperança, Km 04, Dois Vizinhos, Paraná, 85660-0000 Brazil; 2 University of Santa Catarina State, Lages, Santa Catarina, Brazil; 3 Federal University Santa Maria, Santa Maria, Rio Grande Do Sul, Brazil; 4 Coordenation of Forestry Engineering, Federal Technological University of Paraná, Dois Vizinhos, Paraná, Brazil; 5 Federal Technological University of Paraná, Postgraduate Program in Agroecosystems, Dois Vizinhos, Paraná, Brazil

**Keywords:** Terrestrial ecotoxicology, Springtails, Bioindicators, Chronic toxicity, Reproductive impairment, Sublethal effects, Non-target organisms

## Abstract

The agricultural use of pyroligneous extracts has expanded in recent years; however, information regarding their effects on non-target soil organisms remains limited, particularly under tropical conditions. This study evaluated the acute and chronic toxicity of a commercial pyroligneous extract to the springtail *Folsomia candida* in Tropical Artificial Soil. Standardized bioassays were conducted using eight concentrations (0, 0.1, 0.2, 0.4, 1, 2, 4, and 6%). Survival and reproduction were assessed after 28 days of exposure, and ecotoxicological thresholds were estimated using dose–response analyses. Significant effects on survival were observed at 0.2% and 4.0%; however, mortality did not reach 50% at any tested concentration, preventing the estimation of the LC50. The survival pattern suggested a non-monotonic response. Reproduction was the most sensitive endpoint, showing significant reductions from the lowest tested concentration (0.1%), with an EC50 of 3.39% estimated using a nonlinear logistic model. pyroligneous extract may cause relevant sublethal effects on soil fauna and highlight the importance of reproductive endpoints for environmental risk assessment and the establishment of environmentally safe application thresholds in tropical soils.

## Introduction

Pyroligneous extract is a liquid byproduct resulting from wood pyrolysis (Afsharipour et al. 2024). Its chemical composition may vary according to the biomass source and pyrolysis conditions, such as temperature and carbonization time (Leme et al. 2018; Silva & Ataíde 2019). In general, it is characterized by high acidity and a complex mixture of organic compounds, including phenols, organic acids, aldehydes, ketones, and volatile substances (Ouattara et al. 2023; Fiorio et al. 2025). Pyroligneous extract has gained increasing attention in agriculture because of its potential to enhance plant growth and suppress plant pathogens, weeds, and nematodes (Afsharipour et al. 2024; Fedeli et al. 2022; Bouket et al. 2022; Aguirre et al. 2020; Chu et al. 2022; Farias et al. 2020; Iacomino et al. 2024).

Although its agronomic uses are promising, its application in this sector still lacks solid scientific support, especially regarding possible negative impacts resulting from its use in tropical agricultural soils. Major knowledge gaps remain concerning the definition of environmentally safe application rates and the potential impacts of this product on non-target organisms, including soil biota. This limitation is especially relevant in tropical environments, where high biological activity and rapid organic matter turnover may influence the bioavailability and toxicity of the compounds present in pyroligneous extract (Rodríguez-Seijo et al. 2025; Albert & Bloem 2023).

In this context, soil-dwelling organisms such as *Folsomia candida* Willem, 1902 (Collembola: Isotomidae) are widely used as bioindicators in ecotoxicological tests due to their high sensitivity to different contaminants. Previous studies have shown that exposure of this species to different compounds can adversely affect survival and reproduction, as well as alter gene expression, leading to genotoxic and cytotoxic effects (Giordani et al. 2021; Kovačević et al. 2023; Fernandes et al. 2023; Martin et al. 2023; Ilyaskina et al. 2024). Furthermore, exposure to substances characterized by high acidity and the presence of phenolic compounds may induce physiological alterations associated with oxidative stress, cellular integrity, and enzymatic activity, thereby increasing their toxic potential to sensitive organisms (De Oliveira et al. 2021; Azevedo et al. 2024).

Despite the growing demand for ecotoxicological assays in terrestrial environments, the field still faces important limitations, mainly due to the high number of substances applied to agricultural soils (Ford et al. 2021; Gainer et al. 2022). Recent reviews highlight major data gaps for soil organisms, especially invertebrates and soil communities in tropical environments, despite the widespread use of various inputs in these systems (Albert & Bloem 2023; Correia & Rasteiro 2025; Rodríguez-Seijo et al. 2025).

Thus, this study was aimed at evaluating the acute and chronic toxicity of different concentrations of an organic input based on pyroligneous extract on *Folsomia candida* through a standardized ecotoxicological assay, contributing to the understanding of the potential risks of its use in tropical soils.

## Materials and methods

### Test organism

Springtails of the species *F. candida*, originating from matrices provided by the Soil and Sustainability Laboratory, Ecotoxicology area, of the State University of Santa Catarina, Chapecó Campus, were used. The cultures were maintained under controlled conditions in climate-controlled chambers (BOD at a temperature of 20 ± 2 °C and a photoperiod of 12 h:12 h (light/dark), in accordance with the recommendations of ISO standard 11267 (1999) and remained established for two months before the start of the experiments. The organisms were cultured in a standardized culture medium and fed weekly with dry baker’s yeast.

### Preparation of the culture medium and maintenance of stock cultures

The culture medium used for rearing and maintaining the test organisms was prepared with activated charcoal, with pH between 6 and 7, and slow-setting plaster, with pH around 6.4, mixed in a 1:11 (*v*/*v*) ratio and moistened with 60 to 100 mL of distilled water per 100 g of the mixture (ISO 2008; Oliveira Filho et al. 2018). Stock cultures were maintained in the culture medium in covered plastic containers, with weekly moisture adjustment and the addition of 10 mg of dry baker’s yeast per 50 to 100 springtails (ISO 1999; OECD 2009) .

### Synchronization of organisms

Synchronization of the test organisms was performed to obtain homogeneous populations aged between 10 and 12 days. Approximately 100 adult individuals were transferred to fresh culture medium and kept under control conditions in a climate chamber, with weekly moisture adjustment and the addition of 2 mg of dry baker’s yeast to induce oviposition. After 10 days, the eggs were removed with a moistened brush and transferred to new culture media. The containers were monitored until hatching, and after 12 days, the assays were initiated following ISO 11267 (ISO 1999), ISO 17512 (ISO 2008), and OECD 232 (OECD 2009) protocols.

### Tropical Artificial Soil (TAS)

Tropical Artificial Soil was prepared according to Garcia (2004) and Oliveira Filho et al. (2018), consisting of 75% fine sand, 20% kaolin, and 5% coconut fiber as a substitute for peat, originally described by OECD 207 (1984). The sand was washed three times with distilled water and dried at 105 °C for 24 h; the coconut fiber was dried at 60–65 °C for 48 h and sieved through a 2-mm mesh. The use of TAS increases the ecological relevance of ecotoxicological assays conducted under tropical conditions, as its composition was specifically developed to better represent the characteristics of soils found in these environments while maintaining the level of standardization required for comparisons among studies. The replacement of peat with coconut fiber provides a source of organic matter that is more representative of tropical ecosystems and has been widely adopted in ecotoxicological protocols designed for tropical conditions (Silva and van Gestel 2009).

Soil pH was adjusted to the range of 5.5 to 6.5, according to ISO 15799 (2001). Water Holding Capacity (WHC) was determined following ISO 11465 (1993), and the soil was maintained at 60% of this capacity. For the TAS used, total WHC corresponded to 28.97%. Since the extract is liquid and diluted in distilled water, the calculations for the volume of water added to each container were based on the amount of water used to dilute the pyroligneous extract concentrations.

### Treatments

The pyroligneous extract used in this study was a commercial product obtained from the pyrolysis of eucalyptus wood sourced from commercial plantations licensed by the Forestry Institute of the State of Minas Gerais, Brazil. According to the technical information provided by the manufacturer, the carbonization process is conducted at a final temperature of approximately 450 °C and lasts between 90 and 120 h, depending on the moisture content of the wood and the diameter of the logs. During carbonization, the condensable fraction of the gases is collected and subjected to vacuum distillation to remove tar compounds, resulting in the final commercial product.

Information regarding the chemical composition and analytical characterization of pyroligneous extract was obtained from the manufacturer’s technical documentation. The product was characterized using high-resolution gas chromatography coupled with mass spectrometry (HRGC–MS), and the concentrations of polycyclic aromatic hydrocarbons (PAHs) were periodically monitored by accredited analytical laboratories. The physicochemical properties and chemical composition of the pyroligneous extract used in this study are summarized in Table 1.

**Table 1:** Chemical composition of the pyroligneous extract–based bioinput

Compound	Class	Content (% pp)
Constitution water	–	92.5
Acetic acid	Carboxylic acids	3.75
Formic acid	Carboxylic acids	1.35
Propionic acid	Carboxylic acids	0.30
Phenol	Phenolic compounds	0.08
Cresols	Phenolic compounds	0.20
Guaiacol	Phenolic compounds	0.13
Syringol	Phenolic compounds	0.15
Cyclotene	Lactones	0.05
Maltol	Lactones	0.01
pH	–	2.7

Source: adapted from Rezende and Carazza (2004)

Eight concentrations of a pyroligneous extract-based organic input were tested: T0 (control) (0.0%), T1 (0.1%), T2 (0.2%), T3 (0.4%), T4 (1.0%), T5 (2.0%), T6 (4.0%), and T7 (6.0%). The concentration range was established based on agricultural application rates reported in the literature for pyroligneous extracts and included concentrations compatible with the agronomic use of the product, as well as higher concentrations to characterize the dose–response relationship and determine ecotoxicological thresholds (Leifeld and Walz 2025). Considering the average density of the product (1.03 g mL⁻^1^), the tested concentrations corresponded to 515; 1030; 2060; 5150; 10,300; 20,600; and 30,900 mg kg⁻^1^ dry soil for treatments T1 to T7, respectively.

For treatment preparation, the pyroligneous extract was initially diluted in distilled water and subsequently incorporated into the dry soil through manual homogenization until the solution was evenly distributed throughout the experimental matrix, ensuring homogeneous exposure of the test organisms. Soil moisture was adjusted according to the recommendations of ISO 11267 (1999).

Each treatment consisted of six replicates, totaling 48 experimental units. One additional replicate per treatment was used to measure the final soil pH after the test. The experimental units were randomly distributed within the climate-controlled chamber and periodically rotated throughout the experimental period to minimize potential position effects and reduce experimental bias associated with microenvironmental variations within the chamber.

### Acute and chronic toxicity tests

The toxicity assays were conducted simultaneously, following ISO 11267 (1999). For each experimental unit, 30 g of previously moistened TAS contaminated with the respective concentrations of pyroligneous extract were placed in the containers. Ten juvenile *F. candida* (10–12 days old) were introduced into each container (400 mL) using an entomological pipette.

The organisms were fed with 2 mg of dry baker’s yeast at the beginning of the test and on days 14 and 21. Moisture was adjusted weekly to the initial saturation level. Throughout the test, the containers were kept in a climate-controlled chamber at 20 ± 2 °C with a 12 h:12 h photoperiod for 28 days. The organisms were periodically inspected throughout the exposure period to assess their general condition and verify survival.

At the end of the experimental period, adults and juveniles were counted by adding water to the containers to suspend the organisms, followed by the addition of blue liquid dye to facilitate visualization. For each experimental unit, three images were obtained by photographic recording, and the number of surviving adults and juveniles was counted using ImageJ® software (ImageJ 2004).

Validation criteria were then assessed according to ISO 11267 (1999) and ABNT NBR ISO 11267 (2011): adult mortality must be less than 20%, the number of juveniles must exceed 100 individuals per replicate, and the coefficient of variation must be below 30%.

### Statistical analysis

After test validation, the data were subjected to normality and homogeneity of variance analyses using the Kolmogorov–Smirnov and Bartlett tests, respectively. Differences between treatments and the control were evaluated by one-way analysis of variance (ANOVA), followed by Dunnett’s test (*p* < 0.05). Median lethal concentration (LC_50_) was estimated using PriProbit 1.63 software (Sakuma 1998).

The median effective concentration (EC_50_), defined as the concentration causing a 50% reduction in the reproduction, was estimated by nonlinear regression using a three-parameter logistic model fitted to the individual reproduction data in Statistica v.7.0 (StatSoft 2004). For the fitted model, the curve parameters, their respective 95% confidence intervals (95% CI), and the coefficient of determination (*R*^2^) were estimated.

## Results

### Test validation

The bioassays met all validation criteria established by ISO 11267 (1999) and ABNT NBR ISO 11267 (2011). In the control treatment, adult mortality was 5%, which was below the maximum acceptable limit of 20%. The mean juvenile production was 834.67 individuals per replicate, exceeding the minimum requirement for test validity, while the coefficient of variation (CV) for reproduction was 8.81%, remaining below the maximum acceptable value of 30%.

### Acute toxicity

**Fig. 1:**
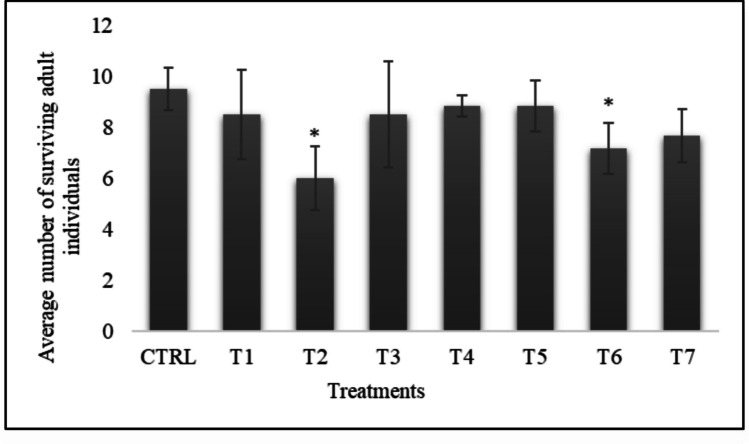
Mean number of surviving adult individuals of *F. candida* after 28 days of exposure to increasing concentrations of pyroligneous extract in Tropical Artificial Soil (TAS). *Significant difference from the control according to Dunnett’s test (*p* < 0.05). T0 = control; T1 = 0.1%; T2 = 0.2%; T3 = 0.4%; T4 = 1.0%; T5 = 2.0%; T6 = 4.0%; and T7 = 6.0%

In the acute toxicity assay, which evaluates lethal concentrations to organisms, a significant difference compared to the control was observed in the 0.2% (T2) and 4.0% (T6) treatments, indicating a toxic effect of pyroligneous extract on the survival of *F. candida* (Fig. 1).

However, the total mortality observed did not allow the calculation of the lethal concentration that caused 50% mortality of adults (LC_50_), due to the absence of a sufficiently defined dose–response curve. The lowest observed effect concentration (LOEC) was 0.2% (T2), while the 0.1% concentration (T1) did not differ from the control and was therefore considered the no observed effect concentration (NOEC) (Table 2).

**Table 2:** Ecotoxicological parameters calculated based on lethality and reproduction tests with *F. candida* exposed to increasing concentrations of pyroligneous extract in Tropical Artificial Soil (TAS)

Observed endpoint	Parameter	Concentration (%)
Lethality (acute toxicity)	NOEC	0.1 (T1)
	LOEC	0.2 (T2)
	LC50	Not determined
Reproduction (chronic toxicity)	LOEC	0.1(T1)
	EC50	3.39 (3.39–46.91)*

*NOEC* concentration corresponding to no observed effects, *LOEC* concentration corresponding to the lowest observed effect, *EC*_*50*_ concentration of the compound at which 50% of the effect on reproduction is observed

*Confidence limits of the estimated values

The mean pH variation between the beginning and the end of the assay was 0.25 units (initial mean pH = 5.64; final mean pH = 5.89).

### Chronic toxicity

**Fig. 2:**
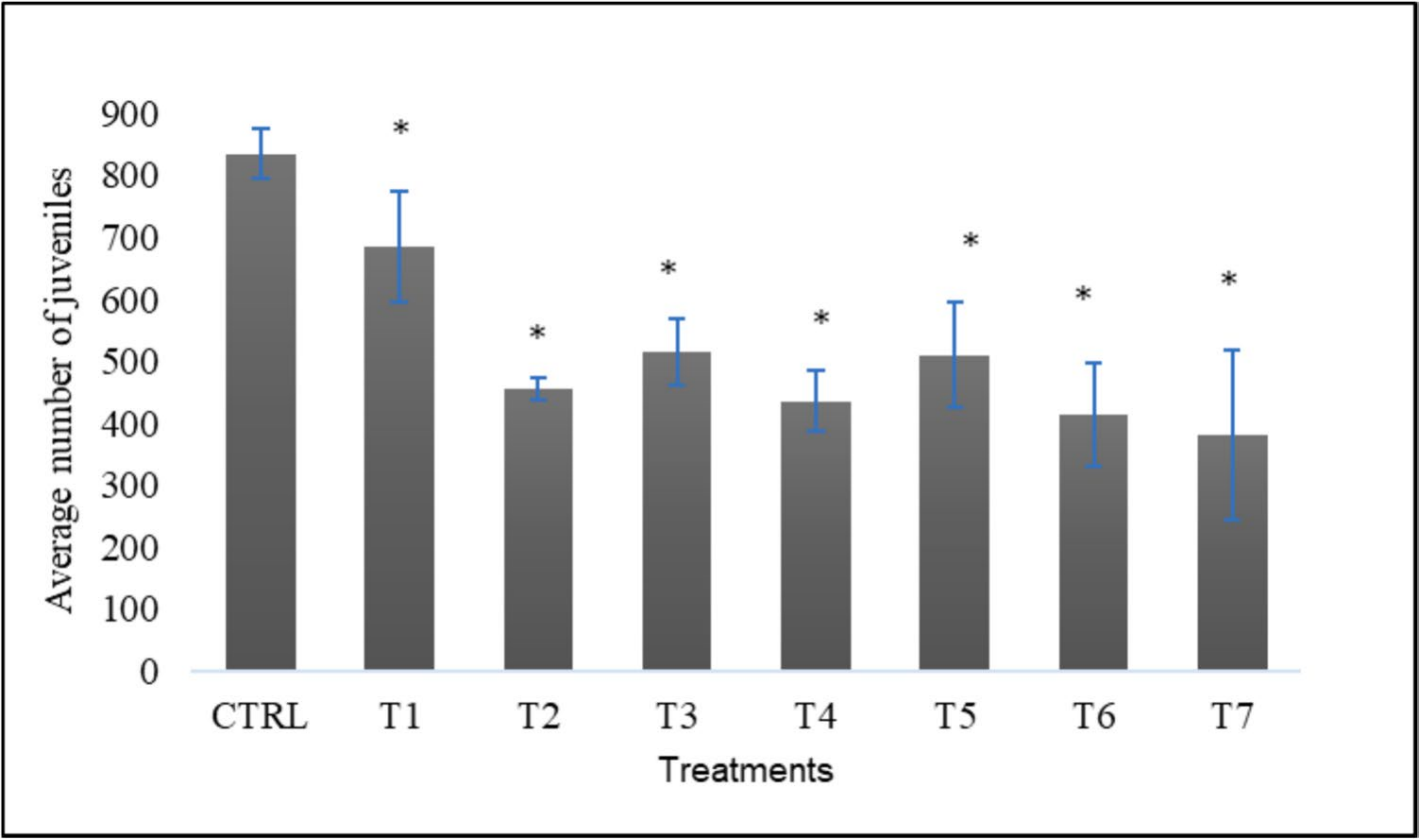
Mean number of *F. candida* juveniles after 28 days of exposure to increasing concentrations of pyroligneous extract in Tropical Artificial Soil (TAS). *Significant difference from the control according to Dunnett’s test (*p* < 0.05). T0 = control; T1 = 0.1%; T2 = 0.2%; T3 = 0.4%; T4 = 1.0%; T5 = 2.0%; T6 = 4.0%; and T7 = 6.0%

In the chronic toxicity assay, all tested concentrations showed a significant reduction in reproduction compared to the control (Fig. 2).

The lowest observed effect concentration (LOEC) for reproduction was 0.1% (T1). The no observed effect concentration (NOEC) could not be established because the lowest concentration tested already produced a statistically significant effect on juvenile production.

The median effective concentration (EC50) for reproduction was estimated at 3.39% (95% CI: 3.39–46.91), indicating a high reproductive sensitivity of *Folsomia candida* to the pyroligneous extract (Table 2). The logistic model explained approximately 68.2% of the observed variability in the data (*R*^2^ = 0.682).

The mean number of juveniles decreased from 834.67 individuals in the control treatment to values ranging from 381.0 to 685.0 individuals in the treatments containing pyroligneous extract.

## Discussion

### Acute toxicity of pyroligneous extract to *F. candida*

Although *Folsomia candida* is known to be sensitive to several abiotic soil factors, including pH (Oliveira Filho and Baretta 2016), the variation observed throughout the experiment remained within the range considered suitable for the development of the species (ISO 2001). Therefore, the slight increase in pH recorded during the assay does not appear sufficient to explain the lethal effects observed. These findings suggest that the acute toxicity detected in this study was primarily associated with the chemical constituents present in the pyroligneous extract. In this context, pH can determine whether active compounds will be available in free or complex form, altering their ability to affect non-target organisms (Maliang et al. 2020).

The survival pattern observed across the concentration gradient suggests a non-monotonic dose–response relationship, characterized by significant mortality at both low and high concentrations, whereas intermediate concentrations did not produce detectable effects. Responses of this nature have been reported for springtails exposed to complex environmental mixtures and natural derived organic compounds, where the magnitude of the biological response does not increase proportionally with exposure concentration (Ardestani & van Gestel 2013; Szabó et al. 2020), such as pyroligneous extract.

This behavior may involve the differential activation of physiological defense mechanism*. Folsomia candida* possesses detoxification systems based on enzymes such as glutathione S-transferases and cytochrome P450 monooxygenases, which can respond to exposure to toxic compounds (Sillapawattana & Schäffer 2017; Zheng et al. 2022). At intermediate concentrations, the induction of these pathways may reduce the fraction of absorbed compounds that remain biologically active, thereby mitigating their toxic effects. In contrast, lower concentrations may be insufficient to trigger adaptive responses, resulting in increased mortality, whereas higher concentrations may exceed the physiological detoxification capacity of the organisms (Benzon et al. 2025). In addition, changes in the chemical bioavailability of the extract at different concentrations may alter its toxic profile and, therefore, generate non-linear dose-dependent responses in soil organisms, contributing to unexpected differences among doses (Benzon et al. 2025).

### Chronic toxicity and effects on reproduction to *F. candida*

The reduction in reproduction observed across all tested concentrations indicates a high reproductive sensitivity of *Folsomia candida* to pyroligneous extract. This effect may be associated with the combined action of its chemical constituents, which are predominantly composed of organic acids, particularly acetic acid, and phenolic compounds (Bennani et al. 2023; Cândido et al. 2023; Iacomino et al. 2024; Ouattara et al. 2024). These constituents represent an important risk factor for *F. candida*, given the well-documented sensitivity of this group to such chemical classes, including reductions in survival and reproduction following exposure to these substances (Fountain and Hopkin 2005).

Similar effects have been reported in other organisms exposed to complex mixtures rich in phenolic compounds. Studies involving *Daphnia magna* have demonstrated toxic effects associated with the presence of these compounds (De Lima et al. 2019), while Mohamed et al. (2022) reported significant reductions in fecundity and development in the spider mite *Tetranychus urticae* following exposure to pyroligneous extract. Natural leachates rich in organic acids and phenolic compounds derived from leaf litter can drastically reduce reproduction and increase mortality in springtails, suggesting that the soluble fraction of these compounds plays a key role in their toxicity (Chomel et al. 2020).

Evidence from other groups of invertebrates further supports this interpretation. Bioassays conducted with the earthworm *Eisenia fetida* have shown low acute toxicity of pyroligneous extract but significant chronic effects on reproduction and cytogenotoxic parameters. These findings suggest that prolonged exposure may impair essential biological processes in different groups of soil fauna, even when direct mortality remains limited (Sivaram et al. 2024).

The biocidal potential of pyroligneous extract has also been widely documented for organisms considered agricultural pests. Pyroligneous extracts derived from palm species promoted immobilization and mortality of the nematode *Scutellonema bradys* at concentrations ranging from 0.75 to 2%, while also reducing the species' reproductive factor by 43% (Farias et al. 2020). Similar results were reported for *Meloidogyne incognita* under field conditions, with approximately 15% reduction in infection following the application of a 1% pyroligneous extract solution (Iacomino et al. 2024). Although these effects are desirable for the control of phytopathogenic organisms, they demonstrate that the same compounds responsible for the pesticidal activity may also affect non-target organisms, including important representatives of the soil fauna.

The results obtained in the present study are consistent with these findings. Although mortality remained relatively low, the pronounced reduction in reproduction indicates significant sublethal effects that may compromise the population dynamics of *Folsomia candida* and, consequently, the ecological functions performed by this group in terrestrial ecosystems.

### Ecotoxicological implications for tropical soils

The results of this study highlight the importance of ecotoxicological assessments of bioinputs intended for agricultural use, particularly in tropical environments, where high biological activity and rapid organic matter turnover may influence the behavior and bioavailability of compounds applied to the soil.

Although pyroligneous extract is recognized for its agronomic benefits, including antimicrobial and nematicidal activity as well as its biostimulant potential, the effects observed on the reproduction of *Folsomia candida* indicate that its application may pose risks to non-target organisms. The occurrence of significant effects at the lowest concentration tested highlights the need for greater caution when establishing environmentally safe application rates.

In this context, *F. candida* proved to be a highly sensitive indicator of alterations caused by pyroligneous extract, reinforcing the relevance of terrestrial ecotoxicological bioassays as a tool to support environmental risk assessment. Future studies conducted under field conditions, involving different soil types and multiple groups of soil fauna, will be essential for establishing safe application thresholds and promoting the sustainable use of pyroligneous extract-based products in tropical agricultural systems.

## Conclusions

Pyroligneous extract exhibited toxic effects on *Folsomia candida*, affecting both survival and, more markedly, reproduction. Although the LC50 could not be estimated due to the low mortality observed, significant effects on survival were detected at specific concentrations.

Reproduction was the most sensitive endpoint, showing significant reductions at the lowest concentration tested (0.1%), with an estimated EC50 of 3.39%. These findings demonstrate that pyroligneous extract may cause relevant sublethal effects on nontarget soil organisms, even at concentrations that do not induce high mortality.

The non-monotonic response pattern observed suggests the involvement of complex interactions between the constituents of the extract and the physiological responses of the organisms, highlighting the need for further studies to elucidate their mechanisms of action and behavior under field conditions.

This study provides novel evidence of the ecotoxicological effects of pyroligneous extract on *F. candida*, contributing to the development of environmental safety criteria and supporting the assessment of the sustainable use of bioinputs in tropical agricultural systems.

## Data Availability

The authors confirm that the data supporting the findings of the current study are available within the article. Further supplementary data is available from the corresponding author upon reasonable request.
